# Clinical characteristics and risk factors of intestinal involvement in Behçet’s syndrome patients: a cross-sectional study from a single center

**DOI:** 10.1186/s13023-021-01772-x

**Published:** 2021-03-17

**Authors:** Cheng-cheng Hou, Jing-fen Ye, Hai-fen Ma, Jian-long Guan

**Affiliations:** Department of Rheumatology and Immunology, Huadong Hospital Affiliated To Fudan University, #221 yan’an west Road, Shanghai, 200040 People’s Republic of China

**Keywords:** Behçet’s syndrome, Intestinal ulcers, Risk factors

## Abstract

**Background:**

Intestinal Behçet’s syndrome (BS) has high morbidity and mortality rates with serious complications. The purpose of this study was to investigate the clinical characteristics and laboratory parameters of intestinal and mucocutaneous BS patients and analyze the risk factors of intestinal involvement in BS patients.

**Methods:**

A retrospective analysis was used to collect the demographic data and laboratory parameters from 97 intestinal and 154 mucocutaneous BS patients. Univariate and multivariate logistic regression analyses were used to investigate the risk factors of intestinal involvement in BS patients.

**Results:**

The most common clinical manifestations of first onset in intestinal BS patients were oral ulceration (100.00%), followed by genital ulcers (62.89%) and erythema nodule (28.87%), gastrointestinal lesions (28.87%), pseudofolliculitis (25.77%), fever (17.53%), arthritis (16.49%), ocular involvement (5.15%), while the least common were vascular involvement (2.06%) and hematologic involvement involvement (2.06%). The most common intestinal segment involved in intestinal BS patients was terminal ileum (30.9%), followed by ileocecal (18.6%), colon (15.5%). By univariate logistic regression analysis, gender, age at hospitalization, age of disease onset, BDCAF, T-SPOT, fever, erythrocyte sedimentation rate (ESR), C-reactive protein (CRP), leukocyte, erythrocyte, hemoglobin (HGB), neutrophil-to-lymphocyte ratio, serum amyloid A, complement 3, albumin, total cholesterol, high-density lipoprotein and interleukin 6 (IL-6) were found all risk factors of intestinal involvement in BS patients (*P* < 0.05 or *P* = 0.00). Moreover, gender (male), BDCAF (≥ 2), ESR (≥ 15 mm/H), CRP (> 10 mg/L), HGB (< 130 g/L) and IL-6 (> 7 pg/ml) were found the independent risk factors of intestinal involvement in BS patients (all *P* < 0.05).

**Conclusions:**

More attention shall be paid to gender, BDCAF, ESR, CRP, HGB and IL-6 in BS patients. When gender (male), BDCAF (≥ 2), ESR (≥ 15 mm/H), CRP (> 10 mg/L), HGB (< 130 g/L) and IL-6 (> 7 pg/ml) being observed, it may reminds that the presence of intestinal involvement in BS patients.

**Supplementary Information:**

The online version contains supplementary material available at 10.1186/s13023-021-01772-x.

## Introduction

Behçet’s syndrome (BS), also known as Behçet’s disease, is a chronic relapsing multisystemic disease, which can cause inflammation of vessels of all size with involvements of several organs and systems [1]. It is characterized by recurrent oral and genital ulcers, ocular lesions, skin manifestations, and arthritis as well as vascular, neurological, and intestinal involvement [2, 3]. BS is also known as the Silk Rout disease with high prevalence in the Mediterranean, the Middle East, and the Far East, and the prevalence is about 14 per 100,000 in China [3].

Intestinal BS is diagnosed when there is a typically shaped ulcer in the gastrointestinal tract and clinical characteristics meet the diagnostic criteria for BS [4, 37]. The frequency of intestinal BS shows a wide variation across countries, ranging from 1 to 50%, being much more common in the Far East compared with the Middle East and Europe [1]. There were few studies on the frequency of intestinal BS in China, and the frequency of intestinal involvement in BS patients was about 17% reported from a small-sized case series [5]. Our previous study revealed that some BS patients having typical ulcers under colonoscopy although they having no gastrointestinal symptoms [6]. Hence, the frequency of intestinal BS in China may be more likely higher than 17%. Recently, our study found that intestinal involvements were the most common major organ involvements in a cohort of 860 BS patients with a prevalence of 20.7% [38]. Furthermore, intestinal BS has high morbidity and mortality rates with serious complications, such as intestinal perforations, fistulas, infections and massive bleeding [7]. Most intestinal BS patients undergo surgery or repeated surgery [8]. However, there were few studies on biomarkers of intestinal involvement in BS patients. Some studies have found elevated C-reactive protein (CRP) levels can predict reactivation and postsurgical relapse of intestinal involvement in BS [1, 8]. In some other studies, interleukin 6 (IL-6) were found significantly higher in BS patients [9–11], which related to clinical activity of BS. Moreover, fecal calprotectin is a useful marker of active gastrointestinal involvement in BS [12]. BS patients who have gastrointestinal symptoms such as hematochezia, bellyache, diarrhea, nausea or vomiting, will undergo colonoscopy to confirm whether they have intestinal involvement. However, in our daily clinical practice, we find some BS patients having typical ulcers under colonoscopy with no gastrointestinal symptoms. Therefore, to find the early biomarkers which can identify whether BS patients complicated with intestinal involvement will prevent serious gastrointestinal complications occurred in BS patients.

Herein, we focused on the clinical characteristics and laboratory parameters in intestinal and mucocutaneous BS patients in China, aiming to investigate the risk factors of intestinal involvement in BS patients.

## Patients and methods

### Patients

**Fig. 1:**
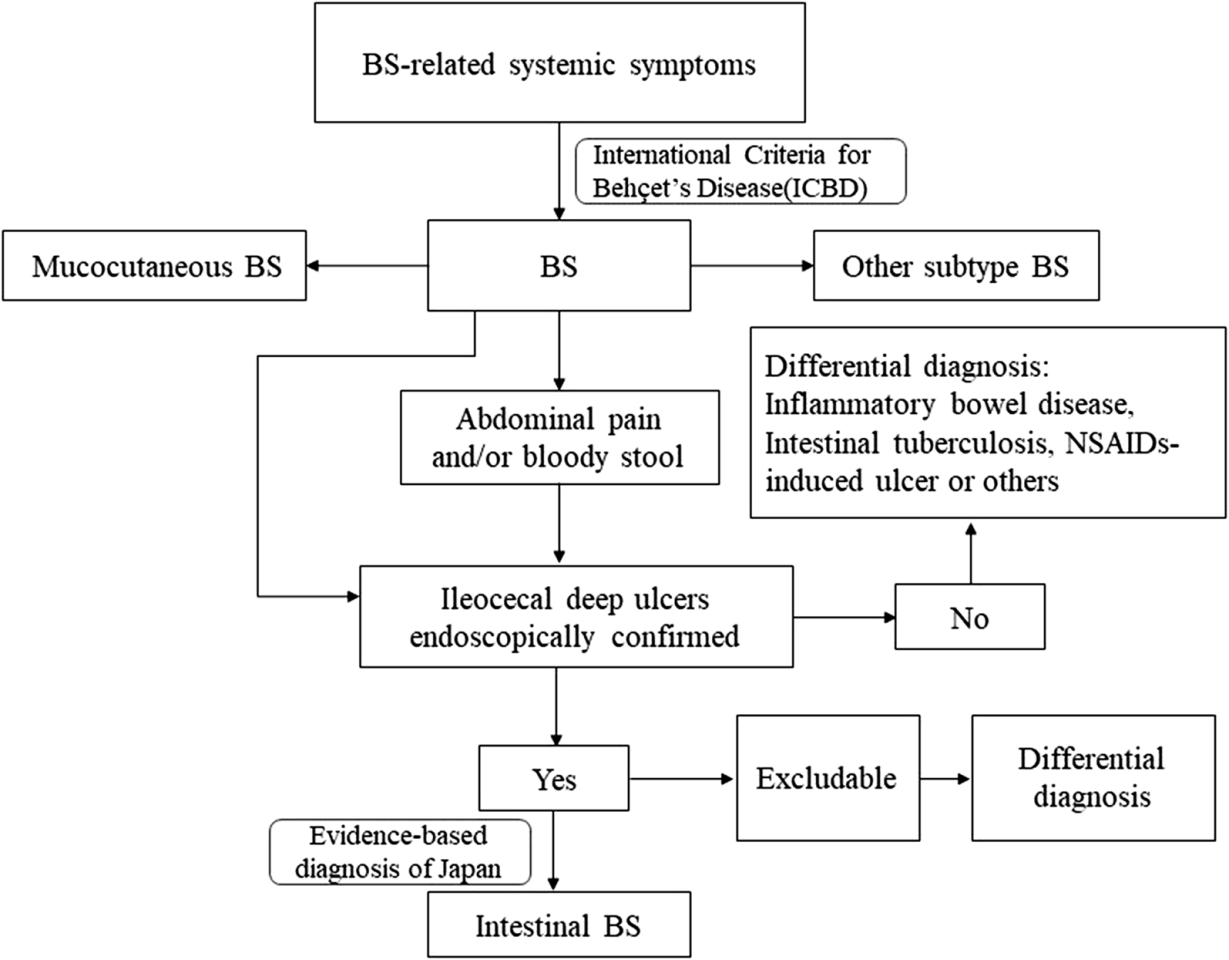
The criteria for inclusion and exclusion of mucocutaneous BS and intestinal BS

A cross-sectional study was performed based on well-organized electronic medical records. We conducted a retrospective analysis of newly diagnosed BS patients who hospitalized in Huadong Hospital affiliated to Fudan University between August 1, 2018 and August 30, 2020 (“newly diagnosed” means a patient diagnosed with BS in the hospital for the first time). All subjects underwent a colonoscopy as part of their routine checkup. The diagnosis of intestinal BS was made in accordance with previously established criteria based on colonoscopic features and clinical manifestations using a modified Delphi process [4, 37] (Fig. 1). Mucocutaneous BS is diagnosed when there are typical mucocutaneous involvements, without damage of intestines, eyes, nervous system, etc. Informed consent was signed by all participants.

Inclusion criteria were as follows: (1) all patients met the new International Criteria for Behçet’s Disease (ICBD) [13]; (2) all patients had complete clinical data and laboratory parameters; (3) all patients were diagnosed with BS in the hospital for the first time. And exclusion criteria were as follows: (1) the patients who were younger than 16 years old; (2) the patients having other disturbing diseases, such as infective diseases, other autoimmune diseases, endocrinal disorders, or malignancies; (3) the patients who had used non-steroidal anti-inflammatory drugs (NSAIDs) in the past 3 months; (4) the patients who were pregnant or lactation women.

## Methods

### Clinical assessment

The clinical manifestations of disease first onset and during hospitalization in BS patients with intestinal involvement and mucocutaneous involvement were retrospectively extracted from the medical records. The disease activity scores of BS patients were recorded using the simplified Behçet’s Disease Current Activity Form (BDCAF) [14]. Patients were interviewed regarding their response to 12 clinical categories over the 4 weeks prior to the day of study enrollment, which composed the frame of BDCAF, and they were then scored from 0 to 12, but only with investigators’ agreements that symptoms were due to BS. Clinical categories were as follows: headache, mouth ulceration, genital ulceration, erythema nodosum, skin pustules, arthralgia, arthritis, nausea or vomiting or abdominal pain, diarrhea or hematochezia, ocular involvement, nervous system involvement and major vessel involvement.

### Data collection

The following information were collected: gender, age, age of disease first onset, disease duration, the score of BDCAF, clinical manifestations of BS, the location of intestinal ulcer, T-SPOT.TB assay (T-SPOT), pathergy test, Hepatitis B core antibody (HBcAb), fever, laboratory parameters (neutrophil-to-lymphocyte ratio (NLR), leukocyte, erythrocyte, hemoglobin (HGB), platelet (PLT), erythrocyte sedimentation rate (ESR), CRP, serum amyloid A (SAA), complement 3 (C3), complement 4 (C4), CH50, albumin, total cholesterol (TCH), triacylglycerol (TG), glucose, uric acid (UA), low-density lipoprotein (LDL), high-density lipoprotein (HDL), immunoglobulin (IgA, IgG, IgE, IgM), IL-6) and treatment approaches.

### Statistical analysis

The software of SPSS version 23.0 (SPSS Inc., Chicago, IL, USA) was used for statistical analysis. Count data were denoted by percentage or ratio. Continuous variables were expressed as mean ± standard deviation (x ± s). Chi-square test or Fisher’s exact test was used for qualitative data analysis. Analysis of variance (ANOVA) was used for quantitative data analysis. Risk factors were analyzed by two classification logistic regression analysis (first by univariate logistic regression analysis to identify significant different variables, then these variables into a multivariate logistic regression analysis selected by forward: LR method) and calculated the odds ratio (OR) and 95% confidence interval (95% CI). Lastly, a Receiving Operating Characteristic (ROC) curve was used to demonstrate the sensitivity and specificity of the selected risk factors. All tests were two-sided. Differences were considered statistically significant when *P* was less than 0.05 (P < 0.05).

## Results

### Clinical manifestations and intestinal lesion sites of intestinal BS patients

A total of 412 newly diagnosed BS patients were recruited in this study. Of the cohort of 412 BS patients, 97 (23.54%) patients were newly diagnosed with intestinal BS, and 154 (37.38%) were mucocutaneous BS. Of 97 intestinal BS patients, 13 (13.40%) patients underwent the surgical treatment of bowel resection. The clinical manifestations of intestinal and mucocutaneous BS patients were shown in Table 1. The most common clinical manifestations of disease first onset in intestinal BS patients were oral ulceration (100.00%), followed by genital ulcers (62.89%) and erythema nodule (28.87%), gastrointestinal lesions (28.87%), pseudofolliculitis (25.77%), fever (17.53%), arthritis (16.49%), ocular involvement (5.15%), while the least common were vascular involvement (2.06%) and hematologic involvement (2.06%). The most common clinical manifestations during hospitalization in intestinal BS patients were nausea/vomiting/abdominal pain (50.52%), followed by oral ulceration (48.45%), diarrhea/hematochezia (23.71%), genital ulcers (16.49%), erythema nodule (14.43%), arthralgia (7.72%), hematologic involvement (6.19%), ocular involvement (3.09%), major vessel involvement (2.06%), arthritis (2.06%), nervous system involvement (1.03%), headache (1.03%), while the least common was skin pustules (0.00%). Other manifestations before or after the intestinal manifestation in intestinal BS patients were shown in Additional files 1. The intestinal lesion sites of 97 patients with intestinal BS were shown in Table 2. The most common intestinal segment involved in intestinal BS patients was terminal ileum (30.9%), followed by ileocecal (18.6%), colon (15.5%), ileocecal and terminal ileum (9.3%), cecum (4.1%), small intestine and ileocecal (3.1%), small intestine and terminal ileum (3.1%), terminal ileum and colon (3.1%), cecum and terminal ileum (2.1%), ileocecal and colon (2.1%), cecum and ileocecal (2.1%), while the least common were small intestine (1.0%), rectum and colon (1.0%), small intestine, colon and cecum (1.0%), small intestine, colon and ileocecal (1.0%), small intestine, terminal ileum and ileocecal (1.0%), ileocecal, colon and rectum (1.0%).

**Table 1:** Clinical manifestations of patients with intestinal BS and mucocutaneous BS

Variables	Intestinal BS (n, %)	Mucocutaneous BS (n, %)
*Clinical manifestations of disease first onset*		
Fever	17 (17.53)	9 (5.84)
Oral ulcers	97 (100.00)	154 (100.00)
Genital ulcers	61 (62.89)	135 (87.66)
Erythema nodule	28 (28.87)	73 (47.40)
Pseudofolliculitis	25 (25.77)	39 (25.32)
Ocular involvement	5 (5.15)	0 (0.00)
Arthritis or Arthralgia	16 (16.49)	21 (13.64)
Vascular involvement	2 (2.06)	0 (0.00)
Gastrointestinal lesions	28 (28.87)	0 (0.00)
Hematologic involvement	2 (2.06)	0 (0.00)
*Clinical manifestations during hospitalization*		
Headache	1 (1.03)	0 (0.00)
Oral ulcers	47 (48.45)	98 (63.64)
Genital ulcers	16 (16.49)	34 (22.08)
Erythema nodule	14 (14.43)	41 (26.62)
Skin pustules	0 (0.00)	2 (1.30)
Arthralgia	7 (7.22)	0 (0)
Arthritis	2 (2.06)	0 (0)
*Nausea/vomiting/abdominal pain*	49 (50.52)	0 (0.00)
Diarrhea/hematochezia	23 (23.71)	0 (0.00)
Ocular involvement	3 (3.09)	0 (0.00)
Nervous system involvement	1 (1.03)	0 (0.00)
Major vessel involvement	2 (2.06)	0 (0.00)
Hematologic involvement	6 (6.19)	0 (0.00)
Surgical treatment of bowel resection	13 (13.40)	–

**Table 2:** Intestinal lesion sites of 97 patients with intestinal BS

Intestinal lesion site	Values, n (%)
Terminal ileum	30 (30.9)
Ileocecal	18 (18.6)
Colon	15 (15.5)
Ileocecal and terminal ileum	9 (9.3)
Cecum	4 (4.1)
Small intestine and ileocecal	3 (3.1)
Small intestine and terminal ileum	3 (3.1)
Terminal ileum and colon	3 (3.1)
Cecum and terminal ileum	2 (2.1)
Ileocecal and colon	2 (2.1)
Cecum and ileocecal	2 (2.1)
Small intestine	1 (1.0)
Rectum and colon	1 (1.0)
Small intestine, colon and cecum	1 (1.0)
Small intestine, colon and ileocecal	1 (1.0)
Small intestine, terminal ileum and ileocecal	1 (1.0)
Ileocecal, colon and rectum	1 (1.0)

### Basic characteristics of 251 BS patients

The demographic variables and laboratory parameters of 97 intestinal BS and 154 mucocutaneous BS patients were shown in Table 3. 49.48% of 97 intestinal BS patients were male, while only 29.22% of 154 mucocutaneous BS patients were male (*P* = 0.00). There was statistically significant difference in terms of gender, age at hospitalization, age of disease onset, BDCAF, T-SPOT, fever, ESR, CRP, erythrocyte, leukocyte, HGB, NLR, SAA, C3, albumin, TCH, HDL, IL-6 between intestinal BS and mucocutaneous BS (all *P* < 0.05). While there was no statistically significant difference (*P* > 0.05) between two groups in terms of disease duration, pathergy test, HBcAb, PLT, IgA, IgE, IgG, IgM, C4, CH50, TG, glucose, UA and LDL.

**Table 3:** Demographic data and laboratory results of BS patients (n = 251)

Variables	Intestinal BS (n = 97)	Mucocutaneous BS (n = 154)	*P*
*Demographic variables*			
Gender (male), n (%)	48 (49.48)	45 (29.22)	0.00*
Age at hospitalization, (years)	34.25 ± 13.14	37.84 ± 13.58	0.04*
Age of disease onset, (years)	26.68 ± 12.26	30.77 ± 12.17	0.01*
Disease duration (years)	7.27 ± 8.30	7.02 ± 7.08	0.80
BDCAF	1.72 ± 0.87	1.16 ± 0.69	0.00*
T-SPOT (+), n (%)	22 (22.68)	18 (11.69)	0.02*
Pathergy test (+), n (%)	43 (44.33)	54 (35.06)	0.14
HBcAb (+), n (%)	26 (26.80)	41 (26.62)	0.98
Fever, n (%)	17 (17.53)	9 (5.84)	0.00*
*Laboratory parameters*			
ESR (mm/H)	35.08 ± 26.16	14.03 ± 14.75	0.00*
CRP (mg/L)	31.18 ± 29.94	7.48 ± 7.81	0.00*
Erythrocyte (10^9/L^)	4.19 ± 0.65	4.34 ± 0.46	0.04*
Leukocyte (10^9/L^)	7.03 ± 2.70	6.18 ± 2.17	0.01*
PLT (10^9/L^)	244.37 ± 89.54	232.62 ± 67.48	0.27
HGB (g/L)	121.21 ± 20.55	129.66 ± 14.61	0.00*
NLR	3.30 ± 2.73	2.13 ± 1.34	0.00*
IgA (g/L)	2.55 ± 1.18	2.72 ± 1.16	0.28
IgE (IU/ML)	102.89 ± 325.37	61.82 ± 108.70	0.23
IgG (g/L)	10.90 ± 3.19	11.38 ± 2.51	0.22
IgM (g/L)	1.29 ± 0.70	1.24 ± 0.58	0.50
SAA (mg/L)	69.60 ± 76.61	17.83 ± 31.37	0.00*
C3 (g/L)	1.26 ± 0.23	1.20 ± 0.19	0.03*
C4 (g/L)	0.25 ± 0.08	0.24 ± 0.07	0.32
CH50 (g/L)	54.12 ± 10.55	53.58 ± 9.38	0.67
Albumin (g/L)	41.86 ± 5.28	43.88 ± 5.13	0.00*
TCH (mmol/L)	4.03 ± 0.84	4.30 ± 1.06	0.03*
TG (mmol/L)	1.24 ± 0.58	1.23 ± 0.65	0.93
Glucose (mmol/L)	4.79 ± 1.41	4.72 ± 0.69	0.58
UA (umol/L)	267.63 ± 80.43	270.42 ± 71.61	0.78
LDL (mmol/L)	2.26 ± 0.63	2.38 ± 0.69	0.17
HDL (mmol/L)	1.34 ± 0.35	1.47 ± 0.34	0.00*
IL-6 (pg/ml)	13.46 ± 17.78	2.70 ± 3.87	0.00*

BDCAF, Behcet’s Disease Current Activity Form; NLR, neutrophil-to-lymphocyte ratio; PLT, platelets; HGB, hemoglobin; SAA, serum amyloid A; C3, complement 3; C4, complement 4; CRP, C-reactive protein; ESR, erythrocyte sedimentation rate; TCH, total cholesterol; TG, triacylglycerol; UA, uric acid; LDL, low-density lipoprotein; HDL, high-density lipoprotein; IL-6, Interleukin 6

*Indicates *P* < 0.05

### Treatment approaches

Our study found that all intestinal BS patients used intravenous steroid during hospitalization while only 18.83% of mucocutaneous BS patients used oral steroid. Most BS patients used thalidomide, and the frequency of mucocutaneous BS patients was higher than that of intestinal BS patients. However, 89.69% of intestinal BS patients used cyclosporine, 69.07% used sulfasalazine, 65.98% used biologics and 10.31% used mesalazine, which were higher than those of mucocutaneous BS patients. Most intestinal BS patients (89.7%) used ≥ 3 immunosuppressants to control disease while most mucocutaneous BS patients (92.9%) used 1 or 2 immunosuppressants. The information of comparison of treatment approaches between intestinal and mucocutaneous BS patients were shown in Table 4.

**Table 4:** Comparison of treatment approaches between intestinal BS and mucocutaneous BS

Variables	Intestinal BS (n = 97, %)	Mucocutaneous BS (n = 154, %)
*Overall treatment*		
Intravenous steroid	97 (100%)	0 (0.00)
Oral steroid	0 (0.00)	29 (18.83)
Sulfasalazine	67 (69.07)	3 (1.95)
Mesalazine	10 (10.31)	0 (0.00)
Colchicine	17 (17.53)	88 (57.14)
Thalidomide	82 (84.54)	143 (92.86)
Total glucosides of paeony capsules	22 (22.68)	108 (70.13)
Hydroxychloroquine	8 (8.25)	21 (13.64)
Cyclosporine	87 (89.69)	79 (51.30)
Biologics	64 (65.98)	4 (2.60)
*Number of immunosuppressants used*		
0	0 (0)	2 (1.3)
1	2 (2.1)	71 (46.1)
2	8 (8.2)	72 (46.8)
≥ 3	87 (89.7)	9 (5.8)

### Analysis of risk factors of intestinal involvement in BD patients

**Fig. 2:**
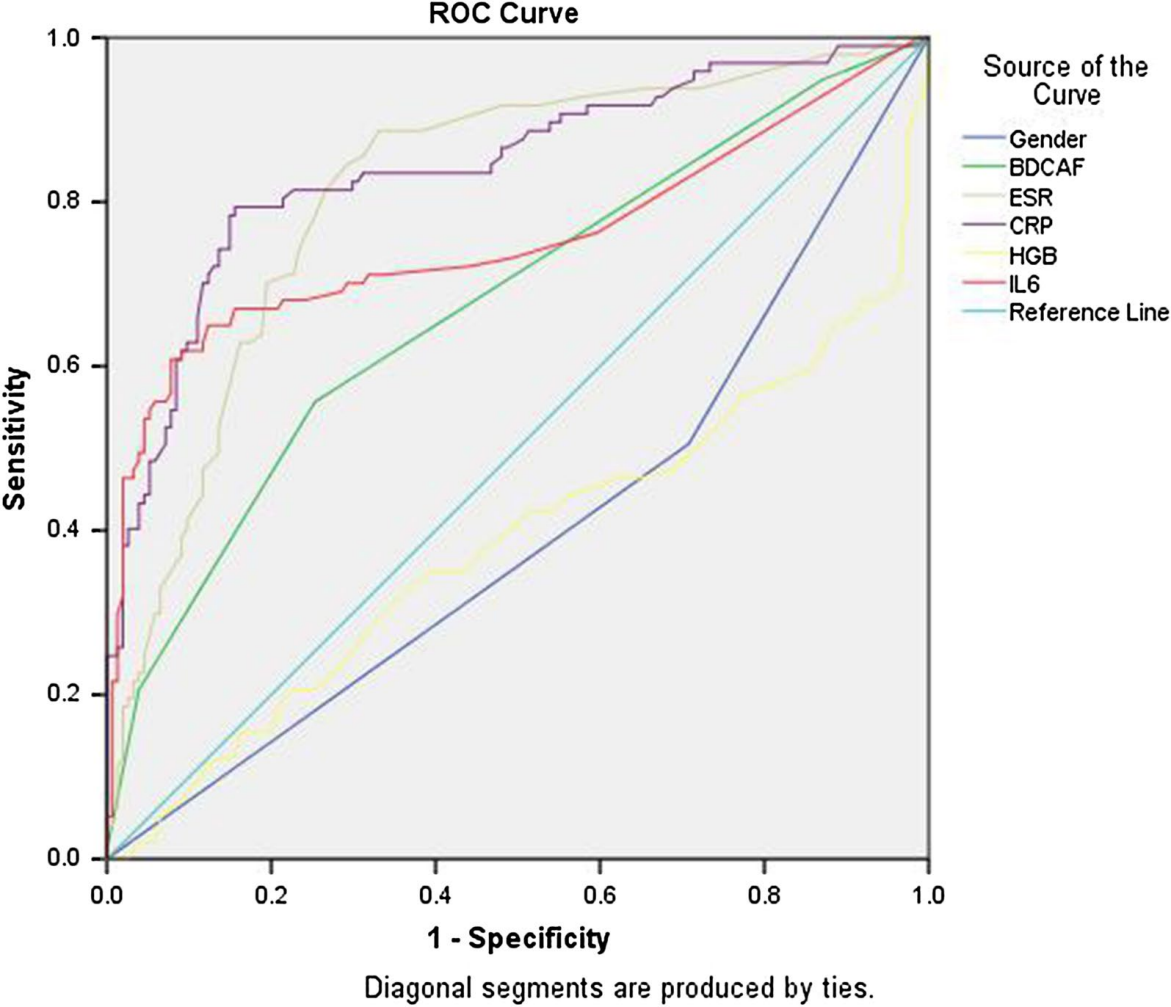
ROC curve analysis. ESR (AUC = 0.814), CRP (AUC = 0.0.843) and IL-6 (AUC = 0.754) could predict whether there was intestinal involvement in mucocutaneous BD patients (*P *= 0.000). However, the gender (AUC = 0.399) and HGB (AUC = 0.384) could not well predict whether there was intestinal involvement in mucocutaneous BD patients (all AUC < 0.5, *P *< 0.05). BDCAF, Behcet’s Disease Current Activity Form; ESR, erythrocyte sedimentation rate; CRP, C-reactive protein; HGB, Hemoglobin; IL-6, Interleukin 6. AUC, area under curve

By univariate logistic regression analysis, gender, age at hospitalization, age of disease first onset, BDCAF, T-SPOT, fever, ESR, CRP, erythrocyte, leukocyte, HGB, NLR, SAA, C3, albumin, TCH, HDL and IL-6 were found all risk factors of intestinal involvement in BS patients (as seen in Table 5). All the above differences were statistically significant (*P* < 0.05 or *P* = 0.00). Additionally, disease duration, pathergy test, HBcAb, PLT, IgA, IgE, IgG, IgM, C4, CH50, TG, glucose, UA and LDL were not correlated with intestinal involvement in BS patients (*P* > 0.05, shown in Table 5). We then selected the meaningful risk factors and turn them into categorical variables including gender (male), age at hospitalization (< 40 years old), age of disease onset (< 30 years old), BDCAF (≥ 2), T-SPOT (+), fever, ESR (≥ 15 mm/H), CRP (> 10 mg/L), erythrocyte (< 4.3*10^9/L^), leukocyte (> 9.5*10^9/L^), HGB (< 130 g/L), NLR (≥ 2), SAA (≥ 10 mg/L), C3 (< 0.8 g/L), albumin (< 35 g/L), TCH (> 5.72 mmol/L), HDL (< 1.29 mmol/L) and IL-6 (> 7 pg/ml) according to clinical or laboratory significance. All the above risk factors were analyzed by multivariate logistic regression analysis. As a result, gender (male), BDCAF (≥ 2), ESR (≥ 15 mm/H), CRP (> 10 mg/L), HGB (< 130 g/L) and IL-6 (> 7 pg/ml) were found the independent risk factors of intestinal involvement in BS patients (as seen in Table 6, all *P* < 0.05). The highest risk rate of BS patients with intestinal involvement was IL-6 (> 7 pg/ml), then followed by the HGB (< 130 g/L), ESR (≥ 15 mm/H), gender (male), CRP (> 10 mg/L) and BDCAF (≥ 2) (shown in Table 6). When the concentration of IL-6 increased (> 7 pg/ml), the risk rate of BS patients with intestinal involvement was 8.23 times more than that with normal level of IL-6. Finally, we performed ROC curve analysis, using gender, BDCAF, ESR, CRP, HGB and IL-6 to predict BS patients with intestinal involvement (Fig. 2). In the ROC curve analysis, we found ESR (AUC = 0.814, 95% CI 0.759–0.869, *P* = 0.000), CRP (AUC = 0.843, 95% CI 0.790–0.896, *P* = 0.000), and IL-6 (AUC = 0.754, 95% CI 0.685–0.824, *P* = 0.000) could predict whether there was intestinal involvement in mucocutaneous BS patients. However, the gender (AUC = 0.399) and HGB (AUC = 0.384) could not well predict whether there was intestinal involvement in mucocutaneous BS patients (all AUC < 0.5, *P* < 0.05).

**Table 5:** Univariate logistic regression analysis of risk factors in BS patients complicated with intestinal involvement

Variable	B	*P*	*OR* (95%CI)
Gender (male), n (%)	0.86	0.00*	2.37 (1.40,4.02)
Age at hospitalization (years)	0.02	0.04*	1.02 (1.00,1.04)
Age of disease onset (years)	0.03	0.01*	1.03 (1.01,1.05)
Disease duration (years)	− 0.00	0.80	1.00 (0.96,1.03)
BDCAF	− 0.92	0.00*	0.40 (0.28,0.57)
T-SPOT (+), n (%)	0.80	0.02*	2.21 (1.12,4.39)
Pathergy test (+), n (%)	0.39	0.14	1.47 (0.88,2.48)
HBcAb (+), n (%)	0.01	0.98	1.01 (0.57,1.79)
Fever, n (%)	1.23	0.00*	3.42 (1.46,8.03)
ESR (mm/H)	− 0.06	0.00*	0.94 (0.92,0.96)
CRP (mg/L)	− 0.11	0.00*	0.89 (0.86,0.92)
Erythrocyte (10^9/L^)	0.52	0.03*	1.68 (1.04,2.71)
Leukocyte (10^9/L^)	− 0.15	0.00*	0.86 (0.78,0.96)
PLT (10^9/L^)	− 0.00	0.24	1.00 (0.99,1.00)
HGB (g/L)	0.03	0.00*	1.03 (1.01,1.05)
NLR	− 0.33	0.00*	0.72 (0.61,0.85)
IgA (g/L)	0.12	0.28	1.13 (0.90,1.42)
IgE (IU/ML)	− 0.00	0.20	1.00 (1.00,1.00)
IgG (g/L)	0.06	0.19	1.07 (0.97,1.17)
IgM (g/L)	− 0.14	0.50	0.87 (0.58,1.30)
SAA (mg/L)	− 0.02	0.00*	0.98 (0.97,0.99)
C3 (g/L)	− 1.37	0.04*	0.26 (0.07,0.91)
C4 (g/L)	− 1.77	0.32	0.17 (0.00,5.45)
CH50 (g/L)	− 0.00	0.67	1.00 (0.97,1.02)
Albumin (g/L)	0.08	0.00*	1.08 (1.03,1.15)
TCH (mmol/L)	0.33	0.03*	1.39 (1.03,1.87)
TG (mmol/L)	− 0.02	0.93	0.98 (0.65,1.48)
Glucose (mmol/L)	− 0.07	0.58	0.93 (0.73,1.19)
UA (umol/L)	0.00	0.77	1.00 (1.00,1.00)
LDL (mmol/L)	0.27	0.17	1.31 (0.89,1.94)
HDL (mmol/L)	1.12	0.00*	3.08 (1.41,6.74)
IL-6 (pg/ml)	− 0.21	0.00*	0.81 (0.75,0.87)

BDCAF, Behcet’s Disease Current Activity Form; NLR, neutrophil-to-lymphocyte ratio; PLT, platelets; HGB, hemoglobin; SAA, serum amyloid A; C3, complement 3; C4, complement 4; CRP, C-reactive protein; ESR, erythrocyte sedimentation rate; TCH, total cholesterol; TG, triacylglycerol; UA, uric acid; LDL, low-density lipoprotein; HDL, high-density lipoprotein; IL-6, Interleukin 6

*Indicates *P* < 0.05

**Table 6:** Multivariate logistic regression analysis of risk factors in BS patients complicated with intestinal involvement

Variable	B	*P*	*OR* (95%CI)
Gender (male)	1.26	0.001*	3.53 (1.65,7.57)
BDCAF (≥ 2)	0.80	0.046*	0.40 (0.28,0.57)
ESR (≥ 15 mm/H)	1.41	0.001*	4.10 (1.75,9.61)
CRP (> 10 mg/L)	1.08	0.017*	2.95 (1.21,7.19)
HGB(< 100 g/L)	1.59	0.038*	4.88 (1.09,21.87)
IL-6 (> 7 pg/ml)	2.11	0.000*	8.23 (2.85,23.76)
Constant	− 5.04	0.000	0.006

BDCAF, Behcet’s Disease Current Activity Form; ESR, erythrocyte sedimentation rate; CRP, C-reactive protein; HGB, Hemoglobin; IL-6, Interleukin 6

*Indicates *P* < 0.05

## Discussion

Intestinal BS is a rare disease, and the epidemiologic studies are scarce. There have been several studies that evaluated clinical outcomes and prognosis of intestinal BS [15, 16]. However, there are few studies on the risk factors of BS complicated with intestinal involvement. Colonoscopy is still the gold standard for diagnosis of intestinal BS, but some patients always refuse to undergo colonoscopy because of its’ invasion and complexity. Herein, we focused on the clinical features and laboratory parameters in intestinal and mucocutaneous BS patients in China, aiming to find the risk factors of intestinal involvement in BS patients. Moreover, our study recruited newly diagnosed patients with intestinal BS and mucocutaneous BS, which can better reflect the difference between the two groups.

Moderate frequency (10%) of intestinal involvement in BS patients in China has been reported [5]. Of the cohort of 412 newly diagnosed BS patients, 23.54% being intestinal BS, which was higher than that reported above and 3.9% reported in Korea [17]. Actually, there were more severe and complicated BS patients gathering in Shanghai for treatment. The higher frequency of intestinal BS in this study may be associated with the above reason. Moreover, many intestinal BS patients without specific gastrointestinal symptoms were diagnosed with intestinal BS by colonoscopy may be also associated with the high frequency. Intestinal manifestations in BS patients usually occur 4.5–6 years after the onset of oral ulcers, and the most frequently involved sites are the terminal ileum [18]. In this study, terminal ileum was also found the most frequently involved sites, which was consistent with the above study. Additionally, our study found that the small intestine was also frequently involved sites with about 10.31% of intestinal BS patients complicated with small intestinal ulcer. We also found that fever and gastrointestinal lesions were more common clinical manifestations of disease first onset in intestinal BS than that in mucocutaneous BS patients.

By univariate logistic regression analysis, we found gender, age at hospitalization, age of disease onset, BDCAF, T-SPOT, fever, ESR, CRP, erythrocyte, leukocyte, HGB, NLR, SAA, C3, albumin, TCH, HDL and IL-6 were all risk factors of BS complicated with intestinal involvement. In terms of gender, some studies revealed that male was a risk factor for eye involvement in BS [19, 20]. Age difference was investigated in studies with relatively small numbers of BS cases. However, those studies failed to show age dependency in most of the manifestations [21, 22]. Our study found age of disease first onset and during hospitalization in intestinal BS patients were younger than those in mucocutaneous BS. Fever, ESR, HGB, NLR and albumin had been demonstrated as indicators that can evaluate disease activity of intestinal BS in a few studies [15, 17, 23–26]. Some studies have found that elevated CRP levels can predict reactivation and postsurgical relapse of intestinal involvement in BS [1, 8, 25, 27]. Additionally, SAA in intestinal BS patients was found significantly higher than that in controls [28], the levels of SAA showed a better correlation with disease activity than CRP [29]. According to clinical or laboratory significance, we selected the above meaningful risk factors and turn them into categorical variables. By multivariate logistic regression analysis, gender (male), BDCAF (≥ 2), ESR (≥ 15 mm/H), CRP (> 10 mg/L), HGB (< 130 g/L) and IL-6 (> 7 pg/ml) were found the independent risk factors of intestinal involvement in BS patients. A few previous studies suggested that there was similar frequency among men and women who complicated with intestinal involvement in BS [20, 30, 31], while several studies showed a slightly increased risk of intestinal BS among males (1.1–1.2:1) [32–34], which was consistent with our finding. This may be related with that some clinical manifestations at initial diagnosis tend to be more severe in male patients and they go to hospital earlier [35]. BDCAF reflects the level of disease activity in BS patients, and it is logical that patients with intestinal involvement have a higher score than those only with mucocutaneous involvement. The elevated levels of ESR and CRP can evaluate disease activity of intestinal BS have been discussed above, and our finding was consistent with the discoveries of other researchers. When ESR ≥ 15 mm/H and CRP > 10 mg/L were found in a mucocutaneous BS patients, the risk of intestinal involvement will increase 4.10 and 2.95 times more than that with normal level of ESR and CRP. Ye JF also found that patients with intestinal BS had lower HGB, higher levels of CRP and higher ESR than those with non-intestinal BS [36], which was consistent with our finding. Several studies have found that IL-6 was significantly higher in BS patients [9–11], suggesting that it was associated with the disease activity of BS. In our study, elevated level of IL-6 (> 7 pg/ml) suggested the risk of intestinal involvement in mucocutaneous BS patients will increase 8.23 times more than those with normal level of IL-6.

Although there are no specific symptoms for intestinal BS, intestinal BS should be considered when abdominal pain, melena/bloody stool, abdominal mass, diarrhea, and weight loss are present. In this study, we found gender (male), BDCAF (≥ 2), ESR (≥ 15 mm/H), CRP (> 10 mg/L), HGB (< 130 g/L) and IL-6 (> 7 pg/ml) were the independent risk factors of intestinal involvement in BS patients. When a BS patient has some gastrointestinal symptoms and the above risk factors, the patient is most likely to be an intestinal BS patient. Colonoscopy should be performed as soon as possible to determine whether there is gastrointestinal damage, and the therapeutic regiment should be adjusted accordingly.

However, our study was conducted in a single center and recruited a relatively small number of BS patients. Our results may not be completely applicable to the general population. Therefore, multi-center samples are needed for verification in the future. Moreover, the risk factors of intestinal involvement in BS patients found in our study were mainly non-specific factors, BS patients with other subtypes may also have the above risk factors. Hence, the above risk factors shall be combined with the gastrointestinal clinical symptoms of BS patients for comprehensive judgment.

## Conclusion

This study investigated the clinical characteristics and laboratory parameters in intestinal BS and mucocutaneous BS patients in China and analyzed the risk factors of intestinal involvement. The independent risk factors of intestinal involvement in BS patients consist of gender (male), BDCAF (≥ 2), ESR (≥ 15 mm/H), CRP (> 10 mg/L), HGB (< 130 g/L) and IL-6 (> 7 pg/ml). When the above factors being observed, it always reminds the presence of intestinal involvement in mucocutaneous BS patients. Hence, higher attention shall be paid to these risk factors and therapeutic regiment shall be adjusted accordingly to avoid the occurrence of serious intestinal complications in BS patients.

## Supplementary Information

**Additional file 1.** Other manifestations before or after the intestinal manifestation in intestinal BS patients.

## Data Availability

The datasets used and analyzed during the current study are available from the corresponding author on reasonable request.

## Abbreviations

BS: Behçet’s syndrome
BDCAF: Behcet’s Disease Current Activity Form
NLR: Neutrophil-to-lymphocyte ratio
PLT: Platelets
HGB: Hemoglobin
SAA: Serum amyloid A
C3: Complement 3
C4: Complement 4
CRP: C reactive protein
ESR: Erythrocyte sedimentation rate
TCH: Total cholesterol
TG: Triacylglycerol
UA: Uric acid
LDL: Low-density lipoprotein
HDL: High-density lipoprotein
IL-6: Interleukin 6
