# Supplementary material for: Clinical characteristics and risk factors of intestinal involvement in Behçet’s syndrome patients: a cross-sectional study from a single center
Source: Orphanet J Rare Dis. 2021 Mar 17;16:132. doi: 10.1186/s13023-021-01772-x (PMC7972242; doi:10.1186/s13023-021-01772-x)
Supplement: Supplementary file 1 — Additional file 1. Other manifestations before or after the intestinal manifestation in intestinal BS patients. [file 13023_2021_1772_MOESM1_ESM.docx]

**Supplementary Table 1 Other manifestations before or after the intestinal manifestation in intestinal BS patients**

| Variables | Before  (n) | At the same time (n) | After  (n) |
| --- | --- | --- | --- |
| Ocular involvement | 5 | 3 | 0 |
| Nervous system involvement | 0 | 0 | 1 |
| Vascular involvement | 2 | 0 | 0 |
| Blood system involvement | 2 | 4 | 0 |
| Arthritis or Arthralgia | 16 | 7 | 0 |
